# Acute selective bioactivity of grape seed proanthocyanidins on enteroendocrine secretions in the gastrointestinal tract

**DOI:** 10.1080/16546628.2017.1321347

**Published:** 2017-06-07

**Authors:** Àngela Casanova-Martí, Joan Serrano, M Teresa Blay, Ximena Terra, Anna Ardévol, Montserrat Pinent

**Affiliations:** ^a^ MoBioFood Research Group. Departament de Bioquímica i Biotecnologia, Universitat Rovira i Virgili, Tarragona, Spain

**Keywords:** Phenolic compounds, ileum, colon, enterohormone, GLP-1

## Abstract

**Background**: Enteroendocrine cells respond to food components by secreting an array of hormones that regulate several functions. We have previously shown that grape seed proanthocyanidins (GSPE) modulate GLP-1 levels.

**Objective**: To deepen on the knowledge of the mechanisms used by GSPE to increase GLP-1, and extend it to its role at modulation of other enterohormones.

**Design**: We used an *ex vivo* system to test direct modulation of enterohormones; STC-1 cells to test pure phenolic compounds; and rats to test the effects at different gastrointestinal segments.

**Results**: GSPE compounds act at several locations along the gastrointestinal tract modulating enterohormone secretion depending on the feeding condition. GSPE directly promotes GLP-1 secretion in the ileum, while unabsorbed/metabolized forms do so in the colon. Such stimulation requires the presence of glucose. GSPE enhanced GIP and reduced CCK secretion; gallic acid could be partly responsible for this effect.

**Conclusions**: The activity of GSPE modulating enterohormone secretion may help to explain its effects on metabolism. GSPE acts through several mechanisms; its compounds and their metabolites are GLP-1 secretagogues in ileum and colon, respectively. *In vivo* GLP-1 secretion might also be mediated by indirect pathways involving modulation of other enterohormones that in turn regulate GLP-1 release.

## Introduction

The intestinal endocrine cells are the largest organ in the human body. They are responsible for the secretion of enterohormones such as glucagon-like peptide-1 (GLP-1) and glucose-dependent insulinotropic peptide (GIP) (together called the incretins), peptide tyrosine tyrosine (PYY), cholecystokinin (CCK), and ghrelin, which are involved in the modulation of food intake, digestion, insulin secretion, and metabolism. New functions for these hormones are still being studied. The modulation of intestinal enterohormone pathways has attracted increasing interest in the fight against widespread pathologies such as obesity and type 2 diabetes. Incretin-based therapies have been established for type 2 diabetes and involve the use of GLP-1 analogues to increase GLP-1 receptor agonist concentrations in the pharmacological range and dipeptidyl peptidase-4 (DPP-4) inhibitors to prevent the degradation of endogenous GLP-1 and GIP, which are both substrates for the DPP-4 enzyme, elevating their plasma levels [1]. The most effective way to increase enterohormones, especially GLP-1 and PYY, is the bariatric bypass known as Roux-en-Y, which is used to treat obesity and also leads to normalization of glucose homeostasis in diabetic patients. GLP-1 secretagogues are therefore in the spotlight as promising therapies against type 2 diabetes and for weight management.

Although studies are being carried out on pharmacological compounds, there are also natural products with capabilities to enhance enterohormone levels, and these could be used as a complementary therapy or in the area of functional foods. They have been recently reviewed [2,3]. Even for these types of product, a complete description of their mechanisms of action is compulsory if their use is to be recommended for a target population. In this regard, grape seed proanthocyanidin extract (GSPE) has been shown to modulate glucose homeostasis and food intake, and this, in part, is mediated through increases in plasma GLP-1 levels [4,5]. GSPE has DPP-4 inhibitory properties, but its effects on GLP-1 secretion need further analysis. *In vivo* studies revealed that increases in plasma GLP-1 levels are only observed after a glucose or meal load, which raises the question of whether GSPE compounds might directly stimulate secretion or modulate the interaction of nutrients with the enteroendocrine cells, for instance making it possible for them to reach more distal parts of the intestine. It has been suggested that the monomeric flavanols found in GSPE bind and activate bitter taste receptors, which, in turn, are regarded as interesting targets to modulate enterohormone secretion.

In this study, we aim to deepen the knowledge of the mechanisms used by GSPE to increase GLP-1 levels, and to determine whether it also modulates other enterohormones.

## Materials and methods

### Materials

GSPE was obtained from Les Dérivés Résiniques et Terpéniques (Dax, France). The same batch (no. 124029) was used in all the studies. According to the manufacturer, the extract contains monomeric (21.3%), dimeric (17.4%), trimeric (16.3%), tetrameric (13.3%), and oligomeric (5–13 U; 31.7%) proanthocyanidins. The small molecules were previously characterized by liquid chromatography–tandem mass spectrometry [6]. A detailed phenolic composition of this GSPE is included in Supplemental Table 1. (–)-Epicatechin (EC) and gallic acid (GA) were obtained from Sigma (St Louis, MO, USA). (–)-Epicatechin gallate (ECg) and procyanidin dimer B2 (B2) were obtained from Extrasynthese (Genay, France). The procyanidin dimer B2–gallate (B2g) was obtained from TransMTT (Gieβen, Germany). For all the studies, stocks were prepared in dimethylsulfoxide (DMSO) and further diluted in the specific buffer required for each experiment.

**Table 1.** Increase in enterohormone concentration in the medium due to grape seed proanthocyanidin extract (GSPE) or gallic acid (GA) treatment in explants from different intestinal segments.

	Duodenum		Ileum	
	GIP	CCK	GLP-1	PYY
GSPE (0.2 mg/ml)	3.32 ± 2.0*	−0.46 ± 0.0*	2.37 ± 0.6*	1.10 ± 0.3*
GA (31 μg/ml)	0.16 ± 0.2	−0.53 ± 0.1*	2.17 ± 1.2	–

Results are expressed as relative units vs control.

GIP, glucose-dependent insulinotropic peptide; CCK, cholecystokinin; GLP-1, glucagon-like peptide-1; PYY, peptide tyrosine tyrosine.

*Statistically significant differences at *p* ≤ 0.05 (Student’s *t* test).

### Cell culture STC-1

The clonal secretin tumour cell line (STC-1) was accepted as a generous gift from Dr B. Wice (Washington University in St Louis, St Louis, MO, USA) with the permission of Dr D. Hanahan (University of California, San Francisco, CA, USA). This enteroendocrine cell line was derived from a double-transgenic mouse tumour [7] and cultured in Dulbecco’s modified Eagle’s medium (DMEM) with GlutaMAX containing 4.5 g/l D-glucose, without sodium pyruvate (Thermo Fisher Scientific, Madrid, Spain), supplemented with 17.5% foetal bovine serum, 100 U/ml penicillin, and 100 mg/l streptomycin (BioWhittaker, Barcelona, Spain), and incubated in a 5% carbon dioxide (CO_2_)-humidified atmosphere at 37°C. Cells were used in passage numbers 30–50.

### Cellular membrane potential of STC-1

To evaluate membrane potential, STC-1 cells were seeded in a 96-well culture plate at a density of 70,000 cells/well for 2 days until they reached 80–90% confluence. The cellular membrane potential (ΔΨcell) was determined in accordance with the method described by Gonzalez-Aubin et al. [8]. In brief, the ΔΨcell was evaluated using fluorescent probe DIBAC4 [3] diluted in 4-(2-hydroxyethyl)-1-piperazineethanesulfonic acid (HEPES) with 10 mM glucose, which was monitored with excitation and emission filters set at 493 nm and 516 nm, respectively. Labelled cells were stimulated with pure compounds and added to final concentrations of 1 μM, 10 μM, 100 μM, and 200 μM.

### Enterohormone release from STC-1

For secretion studies, cells at a density of 2.0 × 10^5^ cells/well were seeded in 24-well culture plates for 2 days to enable 80–90% confluence to be reached. On the day of the experiment, the cells were washed twice with HEPES (20 mM HEPES, 140 mM NaCl, 4.5 mM KCl, 1.2 mM CaCl_2_, and 1.2 mM MgCl_2_ at pH 7.4). Pure phenolic compounds dissolved in HEPES buffer with 10 mM glucose were added to each well, using HEPES buffer 0.05% DMSO as a vehicle. After an incubation period of 2 h at 37°C, supernatants were collected, centrifuged to remove remaining cells, and stored at −80°C until used to determine hormone concentration.

### Enterohormone release from intestinal segments

Rats were killed and their intestines dissected out. For hormone analysis, samples were collected from three different positions along the gastrointestinal tract: the proximal duodenum, distal ileum, and ascending colon. The tissue was rinsed with ice-cold Hank’s balanced salt solution (HBSS; Thermo Fisher Scientific, Madrid, Spain) and dissected into segments (0.75 cm^2^). After a 10 min washing period, tissue segments were placed in prewarmed (37°C) Krebs buffer 0.1% DMSO containing the compounds to be tested with 10 mM glucose or without glucose and 0.1 mM Diprotin A (Enzo Life Sciences International, New York, USA). Duodenal and ileal segments were treated with GSPE or GA and colonic segments were treated with phenolic metabolites from caecal content for 1 h in a humidified incubator at 37°C and 5% CO_2_. To obtain the phenolic metabolites, rats (*n* = 7) were administered a GSPE dose of 500 mg/kg body weight by intragastric gavage 80 min before being killed, and the caecal content was extracted, with the phenolic content of the caecum of non-treated rats being used as a control. The caecal mass (1 g) was dissolved in 10 ml/g phosphate-buffered saline (pH 2) and the phenolic compounds were extracted twice with 10 ml/g ethyl acetate. The organic fraction was nitrogen-dried overnight and reconstituted in 3 ml Krebs buffer 0.1% DMSO for the treatments [9,10].

Tissue viability was checked by the absence of the cytoplasmic marker lactate dehydrogenase (LDH) in the incubated solutions. LDH was analysed using an LDH kit (QCA, Tarragona, Spain).

### Animals and experimental design

Two sets of female Wistar rats, each weighing 180–200 g, were obtained from Harlan (Barcelona, Spain). The subjects were housed singly at 22°C under a 12 h light/dark cycle (lights on at 08:00 h) with access to standard chow pellets (Teklad Global Diets #2014; Harlan, Barcelona, Spain) and tap water *ad libitum* during a 1 week adaptation period. All procedures were approved by the Experimental Animal Ethics Committee of the Universitat Rovira i Virgili.

For the acute treatment in fasting conditions, overnight-fasted female rats (*n* = 5) were treated with 1 g/kg GSPE at the end of the dark period by an oral gavage administration. Vehicle (tap water)-treated rats (*n* = 5) were used as a control group. The abdominal cavity was incised and the portal vein catheterized while body temperature was monitored. Portal blood was obtained at 60 min under sodium pentobarbital anaesthesia before the rats were killed by exsanguination of the aortal vein.

To assess the effects of an acute dose of GSPE in the feeding condition, female rats were treated as previously described [11]. In brief, animals were fasted from 15:00 h to 18:00 h and then treated with 1 g/kg GSPE (*n* = 11) or vehicle (*n* = 10) (tap water) by an oral gavage administration. The animals were then anaesthetized with 70 mg/kg body weight i.p. of sodium pentobarbital and the portal vein was catheterized; at 60 min after the dose, 5 ml mash containing 1.5 g of standard chow and 25 mg of xanthan gum as a stabilizer was punctured into the forestomach with an Abbocath-T 18 G catheter (Hospira, Lake Forest, IL, USA) at a constant rate of 1 ml/min. Portal blood samples were obtained at 80 min from the beginning. After the 120 min procedure, the animals were killed by exsanguination of the aortal vein.

In both models, intestinal segments from the duodenum, jejunum, ileum, and proximal colon were dissected, immediately frozen in liquid nitrogen, and then stored at −80ºC for further enzyme activity and gene expression analysis.

### Enterohormone and glucose quantification

The active GLP-1 concentration from STC-1, intestinal segments, and plasma samples was analysed with a GLP-1 3–37 amide enzyme-linked immunosorbent assay (ELISA) kit (Millipore, Billerica, MA, USA). Total CCK from STC-1 and plasma samples was analysed with a CCK enzyme immunoassay (EIA) kit (Raybiotech, Norcross, GA, USA) and duodenal segments with a CCK8 (desulfated) EIA kit (Peninsula Laboratories, San Carlos, CA, USA). Total GIP levels from duodenal segments were analysed by a total GIP ELISA kit (Millipore, Billerica, MA, USA). PYY from intestinal segments and plasma samples was measured using a fluorescent immunoassay kit (Phoenix Pharmaceuticals, Burlingame, CA, USA). Glucose plasma levels were analysed with an enzymic colorimetric kit (glucose oxidase–peroxidase method; QCA, Tarragona, Spain).

### Measurement of glucose-6-phosphatase enzyme activity from liver and intestine

Liver and intestinal mucosa activities were determined following a modified version of the previously described protocol [12]. Tissues were homogenized in 0.1 M cacodylate buffer (pH 6.5) using a Qiagen Tissuelyser (Qiagen, Hilden, Germany). The suspension was centrifuged and the supernatant incubated in the buffer containing 10 mM glucose-6-phosphate at 37ºC for 20 min. The reaction was stopped at different time-points by adding 100 g/l trichloroacetic acid. Glucose-6-phosphatase (G6Pase) activity was determined by measuring the amount of glucose release from G6Pase using a glucose oxidase–peroxidase coupling system. To assess G6Pase liver activity, the increase in glucose production was measured using an enzymic colorimetric kit (glucose oxidase–peroxidase method; QCA, Tarragona, Spain). G6Pase intestinal activity was assayed following Petrolonis et al. [13], using an Amplex® Red Glucose/Glucose Oxidase Assay Kit (Thermo Fisher Scientific, Barcelona, Spain). Both enzymic activities were normalized per milligram of protein, which was analysed with a BCA Protein Assay Kit (Thermo Fisher Scientific, Barcelona, Spain), using bovine serum albumin as standard.

### Quantitative real-time reverse transcription polymerase chain reaction analysis

Total RNA was extracted using Trizol (Thermo Fisher Scientific, Madrid, Spain) and trichloromethane–ethanol (Panreac, Barcelona, Spain), and purified using a Qiagen RNAeasy kit (Qiagen, Hilden, Germany). The complementary DNA (cDNA) was generated using the High Capacity cDNA Reverse Transcription Kit (Applied Biosystems, Waltham, MA, USA). Quantitative polymerase chain reaction amplification was performed using specific TaqMan probes (Applied Biosystems, Waltham, MA, USA) and the relative expression of each gene was calculated against the control group using the 2-ΔΔCt method, with cyclophilin A (PPIA) as the reference.

### Statistical analysis

Results are presented as mean ± SEM. Data were analysed with SPSS (IBM, Chicago, IL, USA). Data from intestinal segments and STC-1 hormones, gene expression, enzyme activity, and glucose plasma levels were analysed by Student’s *t* tests. The dose–response effect of pure compounds on cellular membrane potential was analysed by one-way analysis of variance. Significance was accepted over 5%.

## Results

### GSPE stimulates enterohormone secretion *ex vivo*

The effects of GSPE on enterohormone secretion were tested in an *ex vivo* intestine model. Bearing in mind that the enteroendocrine cell type is specialized in expressing different hormones throughout the intestinal tract, GIP and CCK were studied in duodenal segments and GLP-1 and PYY in ileum and colon segments [14]. GSPE showed a selective effect at stimulating enterohormone secretion, since in duodenum explants 0.2 mg GSPE/ml (0.17 mg phenolics/ml) increased GIP but decreased CCK secretion (Table 1). In ileum explants the same GSPE dose significantly increased secretion of GLP-1 and, to a lesser extent, that of PYY (Table 1). Lower concentrations of GSPE were also tested and showed no stimulation of GLP-1 secretion (values normalized to those of controls: 1.00 ± 0.08, 1.14 ± 0.12, and 1.22 ± 0.32 for control, 0.1 mg GSPE/ml, and 0.08 mg GSPE/ml, respectively).

In the gastrointestinal tract GSPE is partially absorbed and metabolized, so digested GSPE was used to evaluate the effect at the colon. Proximal colonic sections were incubated for 1 h with non-absorbed phenolic metabolites obtained from the caecal content of animals treated with GSPE and the caecal content of non-treated rats as control. The phenolic concentrations of treated and control colon sections were 188.44 ± 7.02 mg phenolics/l and 88.76 ± 20.64 mg phenolics/l, respectively. The caecal content of GSPE-treated rats enhanced GLP-1 secretion and tended to increase PYY levels (Table 2).

**Table 2.** Glucagon-like peptide-1 (GLP-1) and peptide tyrosine tyrosine (PYY) secretion to the medium in colon explants after 1 h treatment with digested grape seed proanthocyanidin extract (GSPE).

	GLP-1	PYY
**Control**	1.00 ± 0.0	1.00 ± 0.0
**Digested GSPE**	1.34 ± 0.1*	1.32 ± 0.2†

Results are expressed as relative units vs control.

*p < 0.05 vs control; †*p* < 0.05 vs control (Student’s *t* test).

**Figure 1.**
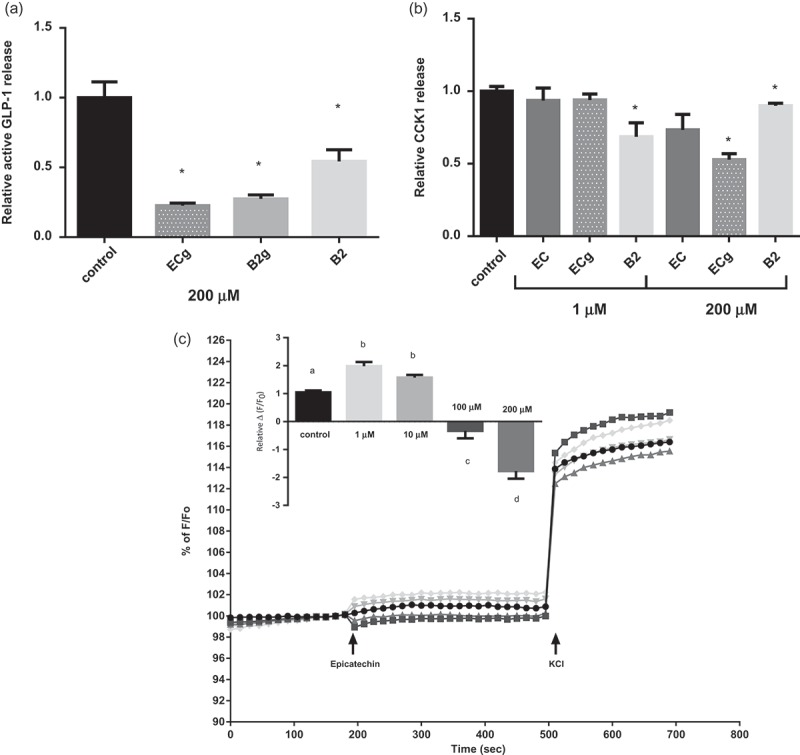
Effects of flavonols on enterohormone secretion and cellular membrane potential in secretin tumour cell line (STC-1) cells. STC-1 cells were treated for 2 h with 200 µM and 1 µM of different flavanols found in grape seed proanthocyanidin extract (GSPE). (a) glucagon-like peptide-1 (GLP-1) and (b) cholecystokinin-1 (CCK1) levels were measured in the culture medium. (c) Effects of flavanols on cellular membrane potential after epicatechin stimulation expressed as % *F*/*F*_0_ and relative Δ(*F*/*F*_0_) (normalized to the control cells) (a), where *F* is fluorescence at 195 s and *F*_0_ is basal fluorescence at 180 s. The data are displayed as the mean ± SEM. *Statistically significant differences versus controls at *p* < 0.05; ^a,b,c,d^statistically significant differences at *p* < 0.05. ECg, (–)-epicatechin gallate; B2g, procyanidin dimer B2–gallate; B2, procyanidin dimer B2.

Since GSPE is a complex mixture of several compounds, we aimed to identify those mainly responsible for the modulation of enteroendocrine secretions working with pure flavanol compounds. Given that some of these compounds could only be obtained in very small amounts, we had to use the STC-1 cell line, which secretes GLP-1 and CCK in a reproducible way [15,16]. The *in vitro* results showed an inhibition of GLP-1 and CCK levels by 200 μM of ECg and B2 (Figure 1). Similarly, the same concentration of B2g decreased GLP-1 secretion, respectively (Figure 1). A lower concentration showed no differences among monomers in the CCK secretion, while a decrease was observed using 1 μM of dimer B2 (Figure 1(b)). The inhibition of hormone secretion by high (200 µM) doses of compounds was in agreement with a cell membrane hyperpolarization found when cell membrane potential was assessed (Figure 1).

GA is a non-flavanol phenolic compound found in significant amounts in grape-seed derived extracts (Supplemental Table 1). Its effects were assayed in the *ex vivo* explants and in STC-1 cells. In *ex vivo* explants, 6.2 µg/ml of GA caused no significant effect in stimulating enterohormone secretion, and this dose is within the range of concentration found in 0.2 mg GSPE/ml. A dose five times higher (31 µg/ml of GA) significantly decreased *in vitro* GLP-1 secretion (values normalized to those of the controls: 1.00 ± 0.10 and 0.72 ± 0.07 for control and 31 µg GA/ml, respectively,  p<0.05). In *ex vivo* explants this dose caused no significant effect on PYY or GLP-1 release, but it inhibited CCK release to the medium by around 50%, similarly to GSPE (Table 1).

### GSPE affects colonic enterohormone gene expression depending on the feeding condition

*In vivo* we previously found that GSPE increases portal active GLP-1 only in the presence of glucose in the intestine [4]. We next analysed how the feeding condition affected GSPE action throughout the intestinal tract by comparing overnight-fasted animals to fed animals. Table 3 shows that 1 g GSPE/kg body weight load in fasted animals led to a reduced expression of GLP-1, PYY, and CCK in the colon. Conversely, the plasma levels of these enterohormones were not significantly changed (Supplemental Table 2).

**Table 3.** Gene expression of the enterohormones from fasted and fed rats.

		Condition	
		Fasted	Fed
GLP-1 colon	Control	1.06 ± 0.2	1.12 ± 0.2
	1 g GSPE/kg bw	0.53 ± 0.0*	1.07 ± 0.2
PYY colon	Control	1.05 ± 0.2	1.13 ± 0.2
	1 g GSPE/kg bw	0.40 ± 0.0*	1.32 ± 0.3
CCK colon	Control	1.01 ± 0.1	1.81 ± 0.8
	1 g GSPE/kg bw	0.36 ± 0.2*	1.09 ± 0.3
CgA colon	Control	1.11 ± 0.3	1.00 ± 0.0
	1 g GSPE/kg bw	2.76 ± 0.5*	2,94 ± 0.5*

Results are expressed as relative units vs control.

GLP-1, glucagon-like peptide-1; PYY, peptide tyrosine tyrosine; CCK, cholecystokinin; CgA, chromogranin A; GSPE, grape seed proanthocyanidin extract; bw, body weight.

*Statistically significant differences at *p* ≤ 0.05 (Student’s *t* test).

In animals that, after a 1 h treatment with GSPE, were administered food for a further 1 h, we found no differences in enterohormone gene expression. In our experimental conditions, there were also no differences in the gene expression of these hormones between the fed and the fasted control animals. As previously published, these animals showed a modified enterohormone plasma profile [5]. We also tested the effects of GSPE in a cell differentiation marker previously shown to be a target for GSPE, chromogranin A (CgA). Table 3 shows that a high acute dose, independently of assay conditions (fed or fasted), increases gene expression.

### Role of glucose in the effect of GSPE’s stimulation of GLP-1 release

Since GSPE increases GLP-1 levels only after feeding (Supplemental Table 2) or an oral glucose load, we next used our *ex vivo* model to analyse whether glucose was required for the direct GSPE stimulation of GLP-1 release. The stimulation of GLP-1 secretion achieved by GSPE in medium containing  glucose 10 mM (shown in Table 1) in medium containing 10 mM glucose was not observed in medium without glucose or with only glucose (1.00 ± 0.1, 1.21 ± 0.1, and 1.17 ± 0.1 in 0 mM glucose, 10 mM glucose, and 0.2 mg of GSPE/ml with 0 mM glucose, respectively; data normalized by 0 mM glucose).

**Figure 2.**
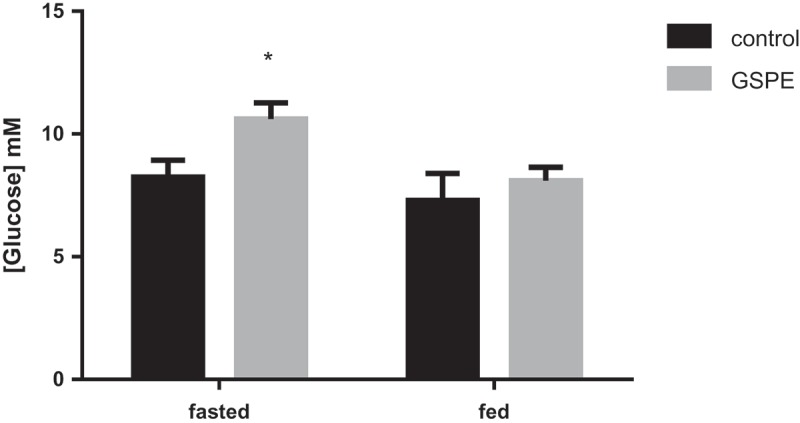
Effects of grape seed proanthocyanidin extract (GSPE) on portal glucose. In the fasted group, overnight-fasted animals were treated with GSPE for 1 h. In the fed group, 4 h-fasted animals were treated with GSPE for 1 h, and then administered a food load. Portal glucose levels were measured 20 min after this food load. *Statistically significant differences versus respective controls at *p* < 0.05.

In our explant system, glucose can reach the cells from either the apical or the basolateral side. To estimate the possible contribution of basolateral glucose presence, we measured portal glucose levels in the fed and fasted animals. Figure 2 shows that portal levels of glucose in overnight-fasted animals treated with GSPE were around 10 mM, while in animals that after 1 h of GSPE treatment were administered a food load, 20 min after this load glucose levels were around 8 mM (significantly different from the fasted animals, *p* ≤ 0.05). In the fed model the glucose levels of GSPE-treated animals did not differ from the controls, while in the fasted animals GSPE significantly increased portal glucose (Figure 2).

Finally, we analysed whether GSPE modulation of portal glucose levels involved modulation of intestinal gluconeogenesis. To do this, G6Pase activity was measured in the duodenal and jejunal sections of fasted and fed animals. Table 4 shows that GSPE inhibits G6Pase activity. In the fasted animals, GSPE inhibited around 60% of intestinal activity (in both measured sections), and in the fed animals this inhibition was stronger, reaching 80% (also in both measured sections). We also measured it in the liver of fed animals and found that there was no change in this enzyme’s activity due to GSPE treatment (1.00 ± 0.1 and 1.02 ± 0.1 in control and treated animals, respectively, data normalized by controls).

**Table 4.** Glucose-6-phosphatase (G6Pase) intestinal activity from fasted and fed rats.

		Condition	
		Fasted	Fed
G6Pase duodenum	Control	1.00 ± 0.2	1.00 ± 0.2
	1 g GSPE/kg bw	0.31 ± 0.0*	0.24 ± 0.0*
G6Pase jejunum	Control	1.00 ± 0.2	1.00 ± 0.2
	1 g GSPE/kg bw	0.45 ± 0.1*	0.21 ± 0.0*

Results are expressed as relative units vs control.

GSPE, grape seed proanthocyanidin extract; bw, body weight.

*Statistically significant differences at *p* ≤ 0.05 (Student’s *t* test).

## Discussion

We have previously shown that GSPE increases plasma active GLP-1 levels, that this is in part due to inhibition of DPP-4 but also to increased secretion, and that this leads to modulation of glucose homeostasis and satiety [4,5]. In this paper, we describe for the first time how GSPE directly modulates enteroendocrine secretions and that it does so differently depending on the intestinal fragment. We also go further into the possible mechanisms by which GSPE exerts such modulation of enterohormone secretion and production.

In this study, we have used an *ex vivo* system to show that GSPE directly stimulates GLP-1 release in the intestine. Direct stimulation could therefore contribute to the previously reported increase in GLP-1 levels [4]. Concerning the mechanisms, we have described that this direct GLP-1 release by GSPE stimulation requires glucose. Furthermore, glucose must be sensed on the luminal side. This agrees with acute *in vivo* results in which, to observe GSPE effects on GLP-1 secretion, either glucose or meal must be added [4,5]. Our results suggest that the effects of GSPE are not secondary effects on glucose absorption and/or metabolism, but direct effects on enteroendocrine cells. A possible explanation for the requirement of glucose could involve a ‘priming’ effect by GSPE of the L-cell in preparation for the subsequent oral glucose-stimulated GLP-1 secretion. This ‘priming effect’ has previously been shown for ghrelin [17] and insulin [18]. Ghrelin pretreatment of GLUTag and NCI-H716 cell lines stimulates GLP-1 release only in a medium containing glucose, similarly to what we observe in GSPE treatments. Furthermore, *in vivo* ghrelin induces GLP-1 release only when an oral glucose load is performed, which is also in concordance with our previous *in vivo* results [4]. Unfortunately, these priming events are still not fully described at the molecular level. Light has only been shed on the requirement of the mitogen-activated protein kinase (MAPK) pathway and MEK-ERK1/2 [17,18], which is a pathway previously shown to be a target for grape seed proanthocyanidins [19].

An unexpected increase in portal glucose levels by GSPE was observed in fasted animals, and we wanted to analyse the role of intestinal gluconeogenesis in this increase. Intestinal gluconeogenesis has been shown to be a mechanism used by different food agents to modulate feeding behaviour [20]. Our results suggest an inhibition of intestinal gluconeogenesis by acute GSPE treatment. The increased portal glucose may therefore be due to reduced glucose uptake by the liver and pancreas, since polyphenols have been previously shown to inhibit the glucose transporter Glut-2 [21,22] and GSPE down-regulates the glucose transporter Glut-2 and glucokinase expression in liver and pancreas [23]. Certainly, considering that glucose may control hunger sensation from the portal vein via signalling to the peripheral neural system [24], increasing portal glucose levels during fasting could, at least in part, contribute to the previously reported [5] satiating effects of GSPE.

GSPE is composed of several different molecules with different bioavailability [25]. Polyphenol absorption in the small intestine is relatively low (5–10%) in comparison to other macronutrients or micronutrients, mainly those with monomeric and dimeric structures [26]. The remaining 90–95% of polyphenols, mainly the polymeric and oligomeric forms, pass through the large intestinal lumen and accumulate in a millimolar range, reaching the colon, where they are subjected to microbial catabolism [27]. As shown in rats treated with the same extract as ours, some final products of colonic metabolites such as 3-*O*-methyl gallic acid and benzoic acids could be detected in the kidneys and liver after 2 h [25], showing that some GSPE compounds, most likely polymeric forms, circulate through the gastrointestinal tract and reach the colon. Our results show that GSPE treatment decreased CCK levels in duodenal segments. We had previously shown that *in vivo* GSPE impaired CCK release after food intake [5]. Inhibition of CCK *ex vivo* is reproduced by GA, a compound found in the extract mixture. The effects of mainly monomeric and dimeric structures of GSPE were also tested in STC-1 cells, and our data show an inhibition of CCK levels. This suggests that molecules that are well absorbed in the upper intestine could be responsible for this direct inhibition. We also found that this inhibition does not lead to a modulation of CCK basal plasma levels in fasted animals (where CCK release is not stimulated). Regarding GLP-1 secretion, which, as previously mentioned, was increased by GSPE in ileum segments, our *in vitro* results in STC-1 cells show that GLP-1 levels are also decreased by structures with a low degree of polymerization. Our results also show that metabolites of digested GSPE promote colonic GLP-1 release. In contrast to CCK secretion, these findings could suggest that unabsorbed polyphenols (high degree of polymerization) and microbiota-metabolized polyphenols of GSPE act on endocrine cells to promote GLP-1 secretion. In agreement with this, a previous study demonstrated that a tetrameric procyanidin increases GLP-1 levels in mice and has more effect on insulin stimulation than smaller procyanidins [28]. Montagut et al. also demonstrated that oligomers can activate insulin signalling and stimulate glucose uptake [19]. Our findings suggest that the absorption and bioavailability of GSPE polyphenols could be involved in enteroendocrine secretion, although further study is needed to understand procyanidin’s effects on enterohormone secretion in more detail.

Another function that may be modulated by GSPE is the inhibition of G6Pase activity, since the levels of inhibition are the same in the different parts of the intestine where it has been measured (i.e. duodenum and jejunum).The inhibition of G6Pase was dependent on the feeding condition. We analysed two animal models that received 1 h GSPE treatment, one killed after an overnight fasting condition and the other fasted for 3 h and then administered a food load in the stomach 60 min after the GSPE dose to determine the effect of feeding. Our results show that inhibition of G6Pase was much stronger in the fed animals. In addition, the feeding condition determined the effects of GSPE at the gene expression level. We found a down-regulation of GLP-1, PYY, and CCK at the colon level in fasted animals treated for 1 h with GSPE. Modulation of gene expression of enterohormones has been studied at the level of intestinal stem cell differentiation [29,30]. Some studies show that the messenger RNA (mRNA) of enterohormones can also be acutely regulated. In rodents, refeeding after a fasting period [31] or feeding a specific nutrient such as palmitoleic acid [32] modulates the mRNA levels of enterohormones within a short time (1–2 h). *In vitro* models have also shown acute modulation of enterohormone gene expression by hormones such as insulin [33,34] and nesfatin [35,36]. In addition, our results show that when a food load was administered after the GSPE, there were no differences between GSPE and controls. This suggests that the effects of nutrients (either directly or mediated by changes in hormones) counteract the down-regulation of GSPE. It should be noted that the fed study was performed after a shorter (3 h) fasting period, so we cannot be sure that GSPE had the same effects on gene expression as in our fasting (overnight) experiment. Certainly, the fasted animals in which down-regulation was observed showed no differences in GLP-1 and CCK plasma levels. At present, we do not know whether such down-regulation at colonic levels might influence enterohormone secretion or if it is involved in the previously mentioned ‘priming’ effect. We also observed that a previously described target of GSPE [37], chromogranin A (CgA), was up-regulated in both conditions. CgA is a marker for enteroendocrine cells [38], but it is unlikely that such short periods of treatment would affect the number of enteroendocrine cells in the colon. There is a lack of information regarding acute regulation of CgA expression in the intestine. However, future work will focus on the effects of GSPE on the differentiation of enteroendocrine cells. GSPE treatment produced a significant induction of other enterohormone secretion in different parts of the intestine. In the duodenum, GSPE directly enhances GIP secretion, which could contribute to *in vivo* GLP-1 secretion due to the enteroendocrine loop between the duodenal GIP and the ileal GLP-1 [39]. In the ileum, GSPE also promotes PYY secretion, to a lesser extent than GLP-1, while a data trend was observed in the colon. It was shown that soy isoflavones enhance PYY secretion in humans [40], although there are few data on the effects of polyphenols on PYY secretion. Altogether, these results reinforce the idea that GSPE has effects throughout the gastrointestinal tract, and that a feeding condition modulates the effects. Further studies are needed to go into greater detail regarding the effects of GSPE effects on the gastrointestinal tract.

In conclusion, the compounds of GSPE act at several points of the gastrointestinal tract modulating enterohormone secretion, which leads to regulation of food intake and glucose homeostasis. The present results suggest that compounds found in GSPE directly promote GLP-1 secretion in the ileum, and its metabolites do so in the colon. Such direct stimulation requires activation of glucose-induced GLP-1-releasing pathways. *In vivo* GLP-1 secretion may also be mediated by indirect pathways involving modulation of other enterohormones that, in turn, regulate GLP-1 release, such as enhancing GIP and reducing CCK secretion in the duodenum (the latter effect being mediated, at least in part, by GA).

## Supplementary Material

Supplementary_Material_Ardevol.docx
