# Supplementary material for: Acute selective bioactivity of grape seed proanthocyanidins on enteroendocrine secretions in the gastrointestinal tract
Source: Food Nutr Res. 2017 Jun 7;61(1):1321347. doi: 10.1080/16546628.2017.1321347 (PMC5475339; doi:10.1080/16546628.2017.1321347)
Supplement: Supplementary_Material_Ardevol.docx [file zfnr_a_1321347_sm6896.docx]

**Supplementary material**

**Table 1**. Main phenolic compounds of the grape seed phenolic extract (GSPE) used in this study, adapted from (Margalef et al., 2015).

| Compound Concentration (μmol/g) | |
| --- | --- |
| Gallic acid | 182.64 ±0.47 |
| Protocatechuic acid | 8.69 ±0.13 |
| Vanillic acid | 4.58 ±0.24 |
| (+)-Catechin | 417.96 ±11.75 |
| (-)-Epicatechin | 321.91 ±14.71 |
| Epicatechin gallate | 47.57 ±2.44 |
| Epigallocatechin | 0.88 ±0.10 |
| Epigallocatechin gallate | 0.07 ±0.00 |
| Procyanidin dimer B1 | 153.50 ±5.98 |
| Procyanidin dimer B2 | 57.46 ±2.40 |
| Procyanidin dimer B3 | 209.71 ±5.89 |
| Gallated dimers | 12.13 ±0.19 |
| Trimers | 6.65 ±0.54 |

**Table 2.** Portal hormone levels 60 min after the GSPE gavage

| hormone | Control | 1g GSPE/Kg bw |
| --- | --- | --- |
| GLP-1 (pmol/L) | 1.80 ± 0.33 | 2.72 ± 0.77 |
| PYY (μg/L) | 0.14 ± 0.07 | 0.09 ± 0.04 |
| CCK (μg/L) | 0.52 ± 0.05 | 0.53 ± 0.07 |

No significant differences, assessed by t-test (p < 0.05) were found between control and GSPE.

**References:**

Margalef, M., Pons, Z., Iglesias-Carres, L., Bravo, F. I., Muguerza, B., & Arola-Arnal, A. (2015). Lack of Tissue Accumulation of Grape Seed Flavanols after Daily Long-Term Administration in Healthy and Cafeteria-Diet Obese Rats. *Journal of Agricultural and Food Chemistry*, *63*(45), 9996–10003. http://doi.org/10.1021/acs.jafc.5b03856
